# MIS-C-Implications for the Pediatric Surgeon: An Algorithm for Differential Diagnostic Considerations

**DOI:** 10.3390/children8080712

**Published:** 2021-08-19

**Authors:** Nora Manz, Claudia Höfele-Behrendt, Julia Bielicki, Hanna Schmid, Matthias S. Matter, Isabella Bielicki, Stefan Holland-Cunz, Stephanie J. Gros

**Affiliations:** 1 Department of Pediatric Surgery, University Children’s Hospital Basel, 4056 Basel, Switzerland; nora.manz@ukbb.ch (N.M.); claudia.hoefele-behrendt@ukbb.ch (C.H.-B.); isabella.bielicki@ukbb.ch (I.B.); stefan.holland-cunz@ukbb.ch (S.H.-C.); 2 Department of Clinical Research, University of Basel, 4056 Basel, Switzerland; 3 Department of Infectious Diseases, University Children’s Hospital Basel, 4056 Basel, Switzerland; julia.bielicki@ukbb.ch (J.B.); hanna.schmid@ukbb.ch (H.S.); 4 Institute of Medical Genetics and Pathology, University Hospital of Basel, University of Basel, 4031 Basel, Switzerland; matthias.matter@usb.ch

**Keywords:** multisystem inflammatory syndrome in children, pediatric inflammatory multisystem syndrome temporally associated with SARS-CoV-2, PIMS-TS, pediatric surgery, acute abdomen, differential diagnosis of appendicitis, SARS-CoV-2

## Abstract

Background: multisystem inflammatory syndrome in children (MIS-C) is a new disease associated with a recent infection with severe acute respiratory syndrome coronavirus type 2 (SARS-CoV-2). Affected children can present predominantly with abdominal symptoms, fever and high inflammatory parameters that might lead to a consult by the pediatric surgeon and an indication for surgery. Methods: clinical data of three patients with MIS-C that underwent surgery were collected. Histopathological analysis of the appendix was performed. Results: we present the clinical course of three children with fever, abdominal pain and vomiting for several days. Clinical examination and highly elevated inflammation markers led to indication for laparoscopy; appendectomy was performed in two patients. Because of intraoperative findings or due to lack of postoperative improvement, all patients were reevaluated and tested positive for MIS-C associated laboratory parameters and were subsequently treated with corticosteroids, intravenous immunoglobulins, acetyl salicylic acid and/or light molecular weight heparin. Conclusions: we discuss the implications of MIS-C as a new differential diagnosis and stress the importance of assessing the previous medical history, identifying patterns of symptoms and critically surveilling the clinical course. We implemented an algorithm for pediatric surgeons to consider MIS-C as a differential diagnosis for acute abdomen that can be integrated into the surgical workflow.

## 1. Introduction

Multisystem inflammatory syndrome in children (MIS-C) is a new disease associated with a recent and sometimes even inapparent infection with SARS-CoV-2 (severe acute respiratory syndrome coronavirus type 2) and has first been described in May 2020 [1,2,3]. Children and adolescents make up a small proportion of coronavirus disease 2019 (COVID-19) cases. National statistics from countries in Asia, Europe and North America show that pediatric cases account for 1–8% of confirmed COVID-19 cases [4,5,6,7]. In the current COVID-19 pandemic, increased incidence of an inflammatory illness occurring in children has been observed. Most cases were reported four to six weeks after the peak of SARS-CoV-2 infections in the affected population [8,9]. The described multisystem inflammation shows symptoms occurring as a result of a cytokine storm affecting different organs, predominantly the gastrointestinal tract, heart, hematological system as well as skin and mucosa [10,11]. In contrast to severe acute COVID-19, pulmonary affection is not frequently described in MIS-C [10,12]. If MIS-C is the suspected diagnosis, treatment recommendations involve surveillance in the intensive care unit, administration of steroids and/or intravenous immunoglobulins, if refractory to therapy even IL-1 receptor antagonists such as anakinra, and thrombosis prophylaxis. Overall mortality seems to be low [8,12,13,14,15,16,17,18].

Preliminary disease definitions have been published in May 2020 by the World Health Organization (WHO), the Centers for Disease Control and Prevention (CDC) and the Royal College of Pediatrics and Child Health [19,20,21]. All definitions include persistent fever, elevated inflammatory parameters, rash and/or bilateral conjunctivitis and/or mucocutaneous lesions, hypotension or shock, laboratory and echocardiographic findings of cardiac dysfunction, evidence of coagulopathy, diarrhea, vomiting, and abdominal pain. Other differential diagnoses should be excluded. Infection with SARS-CoV-2 can be proven either by reverse transcriptase polymerase chain reaction (RT-PCR), serology, antigen test, or suspected by epidemiological link.

In the pediatric emergency room, children with severe abdominal symptoms represent a challenge for both the pediatrician and the pediatric surgeon. In children presenting with an acute abdomen a laparoscopy might be necessary to exclude surgical causes such as perforated appendicitis. Imaging in children with abdominal symptoms and MIS-C by both sonography and CT (computed tomography) scan cannot always clearly differentiate between MIS-C and appendicitis, since appendiceal thickening and ascites have been found in several patients [22]. Due to frequently combined gastrointestinal and cardiac involvement in MIS-C, a recent study suggests that pediatric surgeons preliminarily assess possible cardiac involvement by both laboratory parameters and echocardiography to confirm differential diagnosis of MIS-C in contrast to appendicitis [23].

Although abdominal ultrasound is routinely performed in most centers for suspected appendicitis, ultimate decision-making about laparoscopy still is clinical. Pediatric surgeons should therefore carefully consider MIS-C as a new differential diagnosis in children with acute abdomens.

We present a case series of MIS-C in children that initially presented with abdominal symptoms and were treated surgically, but subsequently developed additional organ involvement typical of MIS-C. In order to intergrade this novel differential diagnosis into pediatric surgical evaluation of patients presenting with abdominal symptoms, we propose an algorithm to support surgical workflow.

## 2. Methods

Case series of 3 pediatric patients admitted at the University Children’s Hospital Basel between November 2020 and February 2021. All patients underwent surgery due to suspected appendicitis and were eventually diagnosed with MIS-C. SARS-CoV-2 detection was performed according to our hospital’s guidelines with RT-PCR (Roche-Cobas-SARS-CoV-2-Target1/Target2, cobas^®^6800) and serological testing by electrochemiluminescence immunoassay (ECLIA) with normal ranges according to the manufacturer.

The appendix was resected in the course of regular treatment and forwarded to the institute of pathology for histological analysis to confirm diagnosis of appendicitis and exclude malignancy. Hematoxylin Eosin staining was performed according to the pathological standard. Secondarily, the appendix was used for further scientific analysis after written patient consent, as approved by the local ethics committee (EKNZ 2015-263).

## 3. Results 

We report the history of three school aged children with MIS-C. All initially presented with abdominal symptoms and underwent exploratory laparoscopy. The clinical characteristics of the described patients are summarized in Table 1 and matched with the defining symptoms of the three preliminary MIS-C complex definitions.

### 3.1. Patient 1

A 9-year-old boy was admitted with a 5-day history of fever, vomiting, constipation and abdominal pain. Contact and travel history did not include any relevant features. On initial examination, he had stable vital signs, was afebrile, but in a reduced general state with a distended abdomen and severe abdominal tenderness. Chapped lips without conjunctivitis and a maculopapular exanthema were described by the parents.

Laboratory work-up showed high inflammatory markers with CRP > 200 mg/L (<10 mg/L) and ESR 68/h (<10 mm/h), low white blood cell count of 5 G/L (4.5–13.5 G/L) with lymphopenia of 0.51 (1.5–6.8), low platelets of 96 G/L (150–450 G/L) and abnormal coagulation parameters with INR 1.3 (0.9–1.1) and fibrinogen of 8 g/L (1.7–4.1 g/L). He showed a hyponatremia of 130 mmol/L (132–145 mmol/L), low chloride (89 mmol/L; (97–107 mmol/L)) and phosphate levels (0.89 mmol/L; (0.95–1.75 mmol/L)), hypoalbuminemia (16 g/L; (35–53 g/L)), and elevated LDH (312 U/L; (<307 U/L)), creatinine (68 µmol/L; (27–54 µmol/L)) and urea (7.3 mmol/L; (2.5–6 mmol/L)). Blood cultures were obtained but remained without bacterial growth. A negative PCR for SARS-CoV-2 from nasopharyngeal swab was obtained upon admission. Sonography showed free fluid and increased enlarged lymph nodes in the right lower quadrant with a thickened wall of the cecum and ascending colon. The appendix could not be visualized.

**Figure 1:**
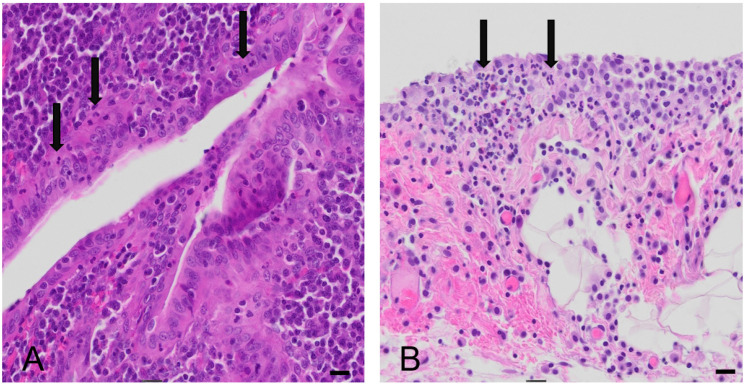
Histomorphological analysis of appendix vermiformis (H&E stain). Presence of acute inflammation in the mucosa of the appendix vermiformis (**A**) and in the periappendiceal fat tissue and serosa of the appendix (**B**). Arrows show neutrophilic granulocytes. Bar equals 50 µm.

Upon pediatric surgical evaluation there was the strong clinical suspicion of a perforated appendicitis with incipient sepsis. Consequently, a diagnostic laparoscopy was performed. Intraoperatively, the patient’s abdominal organs presented with diffuse hyperemia of the entire bowel with free fluid as well as a hyperemic inflamed appendix which was removed. Microbial swabs of the intraabdominal fluid were obtained, and empirical intravenous antibiotic treatment was started. Cultures from the abdominal fluid did not show bacterial growth. The appendix was subjected to histopathological examination, which identified mild signs of acute appendicitis with serositis (Figure 1).

The early post-operative course was atypical of uncomplicated appendicitis, with persisting abdominal pain, inappetence and weakness, low urinary output and high levels of procalcitonin (9.4 µg/L; (<0.1 µg/L)), ferritin (861 µg/L; (14–124 µg/L)), NT-proBNP (n-terminal pro brain natriuretic peptide, 15,844 ng/L; (<125 ng/L)), D-dimers (3.98 µg/mL; (0.19–0.5 µg/mL)) and troponin (128 ng/L; (<14 ng/L)) with normal creatine kinase on extended laboratory work-up. Given increasing suspicion of MIS-C, a SARS-CoV-2 serological evaluation on the 1st postoperative day detected IgM- and IgG-antibodies. In retrospect, the parents reported that the patient had been suffering from afebrile cough and rhinitis without loss of smell or taste 4 weeks previously but was not tested for SARS-CoV-2 at that time.

At the point of diagnosis, the patient was transferred to intensive care due to hypotension and tachycardia. Antibiotic therapy was stopped. Echocardiography on admission to intensive care showed normal cardiac function and no other abnormalities. Steroid therapy with intravenous dexamethasone and thrombosis prophylaxis with low molecular weight heparin was started. The patients’ vital signs normalized within the subsequent 24 h without the need for catecholamines and he improved clinically. After stabilization, the patient was transferred to the pediatric unit. Dexamethasone therapy was stopped after 10 days. Thrombosis prophylaxis was suspended as soon as the patient was sufficiently mobilized. He was discharged in good condition after a hospital stay of 20 days.

### 3.2. Patient 2

A 7-year-old boy who presented to a secondary care hospital initially was transferred to our hospital with a febrile syndrome that included a differential diagnosis of appendicitis and MIS-C. He presented with fever for 3 days as well as vomiting, inappetence and abdominal pain. One month before, he had been diagnosed with a SARS-CoV-2 infection. On clinical examination he was in good general condition, febrile, with stable vital signs, but with diffuse abdominal tenderness.

Abdominal sonography showed enlarged lymph nodes in the lower right quadrant, but the appendix could not be identified. The patient was monitored, with new ascites detected on ultrasound the following day, as well as persistent abdominal pain. Again, the patient underwent exploratory laparoscopy with an intraoperative finding of an inflamed appendix and subsequent appendectomy. Microbiological cultures of the abdominal fluid grew *Bacteroides vulgatus*. Histopathology showed a chronic inflammation with focal lesions of acute inflammation. On the first postoperative day, the patient developed a new maculopapular rash in the right gluteal region, bilateral conjunctivitis and chapped lips.

On account of the unusual pre- and post-operative presentation, an extended laboratory workup was performed despite the intraoperative finding of appendicitis. This confirmed elevated CRP (201 mg/L; (<10 mg/L)), ferritin (362 µg/L; (14–124 µg/L)), D-dimers (5.92 µg/mL; (0.19–0.5 µg/mL)), NT-proBNP (7279 ng/L; (<125 ng/L)), initially normal troponin levels, hypoalbuminemia (18 g/L; (35–50 g/L)) and positive IgG-antibodies for SARS-CoV-2. Echocardiography and sonography did not show any dilated cardiac or abdominal arteries but bilateral basal pleural effusions. Treatment with intravenous immunoglobulins (IVIG), corticosteroids and aspirin were started. Due to increasing levels of NT-proBNP (11993 ng/L; (<125 ng/L)) and newly rising troponin (139 ng/L; (<14 ng/L)), the patient was transferred to our hospital’s intensive care unit. Here, thrombosis prophylaxis was changed from aspirin to low molecular weight heparin, empirical intravenous antibiotic cover was escalated to cover a possible intraabdominal infection, steroid therapy was continued and IVIG was stopped.

After 3 days on the intensive care unit, the patient was sufficiently stabilized and could be transferred to the regular ward. Steroid therapy could be stopped 6 days after first administration and thrombosis prophylaxis after 8 days. He was discharged in good condition 12 days after being first admitted.

### 3.3. Patient 3

An 8-year-old girl presented to the emergency room with abdominal pain for one week, fever for two days as well as diarrhea and vomiting. Her parents had been tested positive for SARS-CoV-2 six weeks prior, but she had not been symptomatic then. She was tachycardic but normotensive and afebrile. Laboratory evaluation showed elevated inflammatory markers with CRP 240 mg/L (<10 mg/L) and leukocytosis of 14 G/L (4.5–13.5 G/L). On clinical examination there was diffuse abdominal tenderness, worst in the right lower quadrant, and discrete guarding. There were no apparent skin or mucosal changes. Sonography showed inflammation of the terminal ileum, free abdominal fluid, lymphadenopathy and fatty tissue imbibition. An appendicitis could not be excluded, and free abdominal fluid suggested possible perforation, so the patient underwent exploratory laparoscopy.

Intraoperatively, the appendix did not show signs of inflammation, the small intestine was mildly dilated and filled with fluid, and small amounts of free turbid fluid were found in the Douglas’ space. No appendectomy was performed. Analysis of the free fluid and of the blood cultures did not show microbial growth. Based on intraoperatively excluded appendicitis and the known SARS-CoV-2 infection of the patient’s parents, laboratory workup was extended and showed normal cardiac parameters, elevated D-dimers (2.9 µg/mL; (0.19–0.5 µg/mL)), fibrinogen (12.7 g/L; (1.7–4.1 g/L)), INR (1.3; (0.9–1.1)), low prothrombin time (59%; (70%–100%)), high ferritin (343 µg/L; (8–79 µg/L)) and hypoalbuminemia (32 g/L; (35–50 g/L)). Serologies for SARS-CoV-2 were positive as was PCR with a low viral load. Clinically, the patient always presented with stable vital signs but with ongoing abdominal pain. She was transferred to the pediatric ward and treated with IVIG and corticosteroids. Due to rising NT-proBNP (575 ng/L; (<125 ng/L)) with normal troponin levels, an echocardiography was performed which was normal. A prophylaxis with low-dose aspirin was initiated. The patient was discharged 5 days after admission in a good state of health with oral corticosteroids for another week and oral aspirin prescribed for 6 weeks.

## 4. Discussion

With regard to the increasing prevalence of children with a history of SARS-CoV-2-infection, MIS-C as a differential diagnosis is of increasing importance for the pediatric surgeon. The three cases clearly demonstrate that MIS-C can present in slightly different variations of the same surgical problem—suspected appendicitis. Patient one suffered from an inflammation of large parts of the intestine, including the appendix. The second patient presented with an isolated appendicitis simultaneously occurring with MIS-C. Additionally, the third patient only suffered from an acute abdomen due to MIS-C without signs of appendicitis. In patient 1, histopathology showed signs of mild appendicitis. Whether a mild appendicitis triggers MIS-C or whether the hyperinflammatory state of children with MIS-C results in infiltration of inflammatory cells in the terminal ileum is not yet understood [24,25].

Next to surgical causes such as acute or even perforated appendicitis, non-surgical problems such as gastroenteritis, yersiniosis with lymphadenitis mesenterialis or inflammatory bowel disease, and less common causes such as Kawasaki disease should be taken into consideration in the differential diagnosis of abdominal pain with inflammatory signs. In our patients, there was no histological proof of Yersinia enterocolitica, as usually performed. Due to the findings confirming the differential diagnosis of MIS-C, as well as quick response to adequate treatment, no further search for other causes was carried out.

Appendicitis has been described as the presenting manifestation of Kawasaki disease (KD) [26,27]. Digestive tract involvement is reported in approximately 20–35% of KD cases and has been recognized as a manifestation of a severe form of the disease [26].

MIS-C was first described in May 2020 and typically presents as a hyperinflammatory state with fever, abdominal pain, mucocutaneous lesions, cardiac dysfunction, hypotension and activated clotting system that occur four to six weeks after primary COVID-19 infection [19,20,21]. However, not all of these clinical manifestations need to be apparent at initial presentation to the emergency room. A considerable proportion of children with MIS-C present with abdominal pain as the lead symptom, often accompanied by vomiting and elevated inflammatory markers, and this may result in consults with the pediatric surgeon. Despite the added information that can be gleaned from abdominal ultrasounds and accounting for the reluctance to expose children to radiation and perform computer tomography, an exploratory laparoscopy can be the only definite way to confirm or exclude the differential diagnoses of acute or perforated appendicitis.

Only a few cases of children diagnosed with MIS-C that underwent surgery due to suspected and perforated appendicitis have been reported [28,29]. The most recently described cases were diagnosed with MIS-C due to their clinical characteristics and did not undergo surgery [30]. Regarding primary SARS-CoV-2 infection, few cases of the combined diagnoses of the acute SARS-CoV-2 infection with an acute appendicitis and diffuse intestinal ischemia in children have been described [31,32]. Overall, not enough is known about the effects of MIS-C on the appendix or the effect of abdominal surgery on MIS-C.

**Figure 2:**
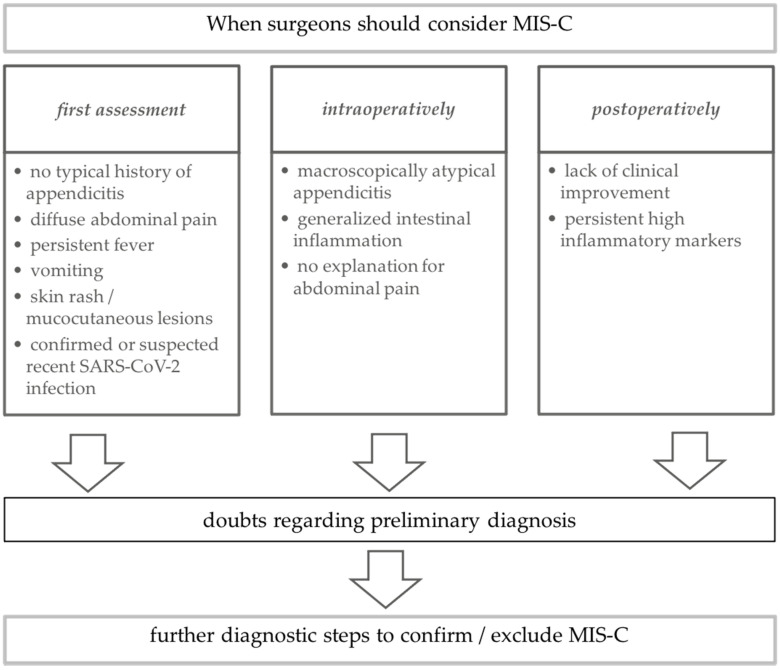
Surgical algorithm for children with typical and atypical abdominal pain regarding differential diagnosis of MIS-C.

As the pandemic proceeds, the share of children with a history of COVID-19 is growing. These may be children who were exposed and not tested, but increasingly also children with a known positive PCR. Our case presentations clearly demonstrate the need for surgeons to consider MIS-C as a differential diagnosis, even when surgery is indicated and indeed when appendicitis is identified on laparoscopy. We therefore suggest an algorithm for the pediatric surgeon to consider MIS-C in the surgical workflow preoperatively, intraoperatively and postoperatively (Figure 2).

Preoperatively, MIS-C should be considered as a differential diagnosis if the child with abdominal pain does not seem to match the typical clinical phenotype of acute or perforated appendicitis; if clinical examination reveals mucocutaneous lesions; if the family reports persistent fever and if a SARS-CoV-2 infection within the last four to six weeks can be confirmed or is strongly suspected. In these children, we suggest performance of additional laboratory tests other than inflammatory parameters, namely coagulation laboratory (d-dimers, PT, PTT, fibrinogen) as well as liver enzymes, ferritin, creatinine and cardiac parameters (NT-proBNP, creatine kinase and troponin).

Intraoperatively, MIS-C should be considered as a differential diagnosis if the findings do not match the macroscopic appearance of an acute or perforated appendicitis, if a generalized gastrointestinal inflammation is seen, or if after careful exploration no reason for the abdominal pain can be found. Postoperatively, MIS-C should be considered as a differential diagnosis if the child does not recover within a reasonable timeframe after surgery or presents with new clinical signs described in the case definitions, such as skin or mucosal abnormalities [19,20,21]. This approach will allow adjustment to include a new differential diagnosis of MIS-C for children presenting with an acute abdomen and will ensure that all necessary surgical and medical management strategies can be implemented at the earliest possible time point.

## Figures and Tables

**Table 1:** MIS-C case-related definitions and clinical patient characteristics of patients 1–3 (P1–3) [19,20,21]. For the diagnosis of MIS-C not all symptoms must be present.

	WHO	CDC	Royal College	P 1	P 2	P 3
Systematic inflammation	fever ≥ 3 days and elevated inflammatory parameters (ESR, CRP, PCT)	fever ≥ 38 °C for ≥ 24 h, elevated inflammatory parameters	persistent fever > 38.5°, lymphadenopathy, elevated inflammatory parameters	yes	yes	yes
Skin and mucosa	rash or bilateral conjunctivitis or mucocutaneous signs	rash, muco-cutaneous lesions	rash, conjunctivitis/swollen hands and feet	yes	yes	no
Fluid balance	hypotension or shock	hypotension or shock	hypotension	yes	no	no
Heart	laboratory or echocardio-graphical findings of myocardial dysfunction, pericarditis, valvulitis, coronary abnormalities	laboratory findings of cardiac dysfunction, myocarditis	laboratory, ECG or echocardiographical findings of myocardial dysfunction, pericarditis, valvulitis, coronary abnormalities	yes	yes	yes
Coagulation	evidence of coagulopathy (PT, PTT, D-dimers)	Elevated d-dimers and fibrinogen	High d-dimers, abnormal fibrinogen	yes	yes	yes
Gastro-intestinal tract	diarrhea, vomiting, abdominal pain	diarrhea, vomiting	diarrhea, vomiting, abdominal pain, ultrasound with colitis, ileitis, lymphadenopathyascites, hepato-splenomegaly	yes	yes	yes
Other organ systems		acute kidney injury		yes	yes	no
	exclusion of microbial cause	no alternative plausible diagnosis	exclusion of microbial cause including toxic shock syndromes	yes	yes	yes
Evidence of SARS-CoV-2	PCR, antigen test, serology or likely contact with SARS-CoV-2 positive person	positive PCR, serology or antigen test or exposure within 4 weeks prior to onset of symptoms	positive SARS-CoV-2-PCR	yes	yes	yes

ESR (erythrocyte sedimentation rate), CRP (C-reactive protein), PCT (procalcitonin), PT (prothrombin time), PTT (partial thromboplastin time).

## Data Availability

All available data is published in manuscript.

## References

[1] Riphagen S., Gomez X., Gonzalez-Martinez C., Wilkinson N., Theocharis P. (2020). Hyperinflammatory shock in children during COVID-19 pandemic. Lancet.

[2] Verdoni L., Mazza A., Gervasoni A., Martelli L., Ruggeri M., Ciuffreda M., Bonanomi E., D’Antiga L. (2020). An outbreak of severe Kawasaki-like disease at the Italian epicentre of the SARS-CoV-2 epidemic: An observational cohort study. Lancet.

[3] Licciardi F., Pruccoli G., Denina M., Parodi E., Taglietto M., Rosati S., Montin D. (2020). SARS-CoV-2-Induced Kawasaki-Like Hyperinflammatory Syndrome: A Novel COVID Phenotype in Children. Pediatrics.

[4] Coronavirus Disease 2019 (COVID-19): Epidemiology Update. https://health-infobase.canada.ca/COVID-19/epidemiological-summary-COVID-19-cases.html.

[5] European Centre for Disease Prevention and Control COVID-19 Situation Dashboard. https://qap.ecdc.europa.eu/public/extensions/COVID-19/COVID-19.html#global-overview-tab.

[6] The Novel Coronavirus Pneumonia Emergency Response Epidemiology Team (2020). The Epidemiological Characteristics of an Outbreak of 2019 Novel Coronavirus Diseases (COVID-19) in China. Zhonghua Liu Xing Bing Xue Za Zhi.

[7] Pakistan Cases Details COVID-19 Dashboard. https://covid.gov.pk/stats/pakistan.

[8] Feldstein L.R., Rose E.B., Horwitz S.M., Collins J.P., Newhams M.M., Son M.B.F., Newburger J.W., Kleinman L.C., Heidemann S.M., Martin A.A. (2020). Multisystem Inflammatory Syndrome in U.S. Children and Adolescents. N. Engl. J. Med..

[9] Belot A., Antona D., Renolleau S., Javouhey E., Hentgen V., Angoulvant F., Delacourt C., Iriart X., Ovaert C., Bader-Meunier B. (2020). SARS-CoV-2-related paediatric inflammatory multisystem syndrome, an epidemiological study, France, 1 March to 17 May 2020. Eurosurveillance.

[10] Jiang L., Tang K., Levin M., Irfan O., Morris S.K., Wilson K., Klein J.D., Bhutta Z.A. (2020). COVID-19 and multisystem inflammatory syndrome in children and adolescents. Lancet Infect. Dis..

[11] Gottlieb M., Bridwell R., Ravera J., Long B. (2021). Multisystem inflammatory syndrome in children with COVID-19. Am. J. Emerg. Med..

[12] Abrams J.Y., Godfred-Cato S.E., Oster M.E., Chow E.J., Koumans E.H., Bryant B., Leung J.W., Belay E.D. (2020). Multisystem Inflammatory Syndrome in Children Associated with Severe Acute Respiratory Syndrome Coronavirus 2: A Systematic Review. J. Pediatrics.

[13] Radia T., Williams N., Agrawal P., Harman K., Weale J., Cook J., Gupta A. (2020). Multi-system inflammatory syndrome in children & adolescents (MIS-C): A systematic review of clinical features and presentation. Paediatr. Respir. Rev..

[14] Henderson L.A., Canna S.W., Friedman K.G., Gorelik M., Lapidus S.K., Bassiri H., Behrens E.M., Ferris A., Kernan K.F., Schulert G.S. (2020). American College of Rheumatology Clinical Guidance for Multisystem Inflammatory Syndrome in Children Associated With SARS–CoV-2 and Hyperinflammation in Pediatric COVID-19: Version 1. Arthritis Rheumatol..

[15] Kaushik A., Gupta S., Sood M., Sharma S., Verma S. (2020). A Systematic Review of Multisystem Inflammatory Syndrome in Children Associated With SARS-CoV-2 Infection. Pediatric Infect. Dis. J..

[16] Aronoff S.C., Hall A., Del Vecchio M.T. (2020). The Natural History of SARS-CoV-2 Related Multisystem Inflammatory Syndrome in Children (MIS-C): A Systematic Review. J. Pediatric Infect. Dis. Soc..

[17] Whittaker E., Bamford A., Kenny J., Kaforou M., Jones C.E., Shah P., Ramnarayan P., Fraisse A., Miller O., Davies P. (2020). Clinical Characteristics of 58 Children With a Pediatric Inflammatory Multisystem Syndrome Temporally Associated with SARS-CoV-2. JAMA.

[18] Cheung E.W., Zachariah P., Gorelik M., Boneparth A., Kernie S.G., Orange J.S., Milner J.D. (2020). Multisystem Inflammatory Syndrome Related to COVID-19 in Previously Healthy Children and Adolescents in New York City. JAMA.

[19] World Health Organization Multisystem Inflammatory Syndrome in Children and Adolescents Temporally Related to COVID-19. https://www.who.int/news-room/commentaries/detail/multisystem-inflammatory-syndrome-in-children-and-adolescents-with-COVID-19.

[20] Centers for Disease Control and Prevention Multisystem Inflammatory Syndrome in Children (MIS-C) Associated with Coronavirus Disease 2019 (COVID-19). https://www.cdc.gov/mis-c/hcp/.

[21] Royal College of Pediatrics and Child Health Guidance: Paediatric Multisystem Inflammatory Syndrome Temporally Associated with COVID-19. https://www.rcpch.ac.uk/resources/paediatric-multisystem-inflammatory-syndrome-temporally-associated-COVID-19-pims-guidance.

[22] Morparia K., Park M.J., Kalyanaraman M., McQueen D., Bergel M., Phatak T. (2021). Abdominal Imaging Findings in Critically Ill Children With Multisystem Inflammatory Syndrome Associated With COVID-19. Pediatric Infect. Dis. J..

[23] Valitutti F., Verde A., Pepe A., Sorrentino E., Veneruso D., Ranucci G., Orlando F., Mastrominico A., Grella M.G., Mandato C. (2021). Multisystem inflammatory syndrome in children. An emerging clinical challenge for pediatric surgeons in the COVID 19 era. J. Pediatric Surg. Case Rep..

[24] Anderson J.E., Campbell J.A., Durowoju L., Greenberg S.L.M., Rice-Townsend S.E., Gow K.W., Avansino J. (2021). COVID-19-associated multisystem inflammatory syndrome in children (MIS-C) presenting as appendicitis with shock. J. Pediatric Surg. Case Rep..

[25] Lishman J., Kohler C., de Vos C., van der Zalm M.M., Itana J., Redfern A., Smit L., Rabie H. (2020). Acute Appendicitis in Multisystem Inflammatory Syndrome in Children With COVID-19. Pediatric Infect. Dis. J..

[26] Garnett G.M., Kimball S., Melish M.E., Thompson K.S., Puapong D.P., Johnson S.M., Woo R.K. (2014). Appendicitis as the presenting manifestation of Kawasaki disease. Pediatric Surg. Int..

[27] Ulloa-Gutierrez R., Gutierrez-Alvarez R., Avila-Aguero M.L. (2004). Kawasaki disease mimicking an acute appendicitis. J. Pediatric.

[28] Harwood R., Partridge R., Minford J., Almond S. (2020). Paediatric abdominal pain in the time of COVID-19: A new diagnostic dilemma. J. Surg. Case Rep..

[29] Jackson R.J., Chavarria H.D., Hacking S.M. (2020). A Case of Multisystem Inflammatory Syndrome in Children Mimicking Acute Appendicitis in a COVID-19 Pandemic Area. Cureus.

[30] Gerall C.D., Duron V.P., Griggs C.L., Kabagambe S.K., Maddocks A.B., DeFazio J.R. (2020). Multisystem Inflammatory Syndrome in Children Mimicking Surgical Pathologies: What Surgeons Need to Know about MIS-C. Ann. Surg..

[31] Meyer J.S., Robinson G., Moonah S., Levin D., McGahren E., Herring K., Poulter M., Waggoner-Fountain L., Shirley D.A. (2021). Acute appendicitis in four children with SARS-CoV-2 infection. J. Pediatric Surg. Case Rep..

[32] Khesrani L.S., Chana K., Sadar F.Z., Dahdouh A., Ladjadj Y., Bouguermouh D. (2020). Intestinal ischemia secondary to COVID-19. J. Pediatric Surg. Case Rep..

